# Preventive effects of hydroalcoholic extract of saffron on hematological parameters of experimental asthmatic rats

**Published:** 2013

**Authors:** Somayyeh Vosooghi, Maryam Mahmoudabady, Ali Neamati, Heydar Aghababa

**Affiliations:** 1 *Department of Biology, PayameNoor University, Boshruyeh, South Khorasan, I. R. Iran*; 2 *Applied Physiology Research Centre and Department of Physiology, School of Medicine, Mashhad University of Medical Sciences, Mashhad, I. R. Iran*; 3 *Department of Biology, Faculty of Science, Mashhad Branch, Islamic Azad University, Mashhad, I. R. Iran*; 4 *Department of Biology, Faculty of Science, Arsanjan Branch, Islamic Azad University, I. R. Iran *

**Keywords:** Asthma, *Crocus sativus*, Inflammation, Rat, Sensitization, WBC

## Abstract

**Objective:** Asthma is a chronic inflammatory disease of the respiratory airways distinguished by edema and infiltration of inflammatory immune cells. To test our hypothesis about the anti-inflammatory effect of saffron, we examined effects of *Crocus sativus (C. sativus*) extract as a prophylactic anti-inflammatory agent in sensitized rats.

**Materials and Methods**: To induce experimental asthma, rats were sensitized with injection and inhalation of ovalbumin (OA). Forty male Wistar rats were divided into 5 groups (n=8 for each): control, sensitized (asthma), and sensitized and pretreated with three different concentrations of extract, 50, 100, and 200 mg/kg, 2 times a week (group asthma+50EX, group asthma+100EX, and group asthma+200EX). After 32 days, total white blood cells (WBC) counts, red blood cells (RBC), and platelet counts in blood were examined.

**Results:** Total WBC number and eosinophil and neutrophil percentage in blood were increased, but lymphocyte decreased in sensitized animals compared with those of control group (p<0.05 to p<0.001). We observed also elevated levels in RBC and platelet counts after sensitization in the asthma group. Pretreatment of sensitized rats in all concentrations decreased WBC count which was significant in first two concentrations (p<0.01 compared with group asthma). All concentrations of extract decreased eosinophil percentage significantly (p<0.001 compared with group asthma), however, for neutrophil percentage this improvement was not significant. Lymphocyte percentage increased in group asthma+100EX compared with group asthma (p<0.05). Moreover, in all concentrations, the extract reduced RBC and platelet count in pretreated sensitized rats compared with group asthma (p<0.01 to p<0.001).

**Conclusion:** Our findings indicated that the extract of *C. sativus* could be useful to prevent asthma as an anti-inflammatory treatment.

## Introduction

Bronchial asthma is a chronic disorder characterized by airway inflammation, reversible airway obstruction, mucus hypersecretion, and airway hyper-responsiveness (Nakagome and Nagata, 2011 ▶). In the process of airway inflammation of asthma, a variety of cells, such as eosinophils, lymphocytes, mast cells, and neutrophils are involved. In susceptible individuals, this inflammation causes recurrent episodes of wheezing, breathlessness, chest tightness, and coughing (Bloemen et al., 2007 ▶). Eosinophils preferentially accumulate at sites of allergic inflammation and are believed to play an important role in the pathophysiology of asthma through the release of a variety of inflammatory mediators, including radical oxygen species and cytokines (Gleich, 2000 ▶). Accumulating evidence established that eosinophils largely contribute to the development of airway remodeling of asthma through transforming growth factor (TGF)-β (Flood-Page et al., 2003 ▶; Humbles et al., 2004 ▶). The other possible mechanism by which eosinophils contribute to the airway disease of asthma may be by adhesion to and then migrating across vascular endothelial cells. These processes are largely regulated by cytokines/chemokines produced by a variety of cells, including T-helper 2 (Th2) cells (Barrett and Austen, 2009 ▶). Not only eosinophilic inflammation but also neutrophilic inflammation may play roles in severe asthma, so that infiltration of these two cell types is increased into the airways of asthmatic patients. Since many inﬂammatory cells play a role in the pathological characteristic feature of asthma, anti-inflammatory treatment is a main solution for management of the disease. 

Crocus sativus L. is a flowering plant in the Iridaceae family and is commonly known as saffron (Hosseinzadeh and Nassiri-Asl, 2012 ▶). Different constituents of the stigma of this plant are crocins, safranal, picrocrocin, ketoisophorone, isophorone, and glycosidic terpenoids (Tarantilis et al., 1995 ▶).

In traditional medicine, the stigmas of this plant have been used as antispasmodic and expectorant (Rios et al., 1996 ▶). Pharmacological studies proved that saffron extracts or its compounds have radical scavenging and anti-oxidant properties (Assimopoulou et al., 2005 ▶; Rios et al., 1996 ▶). Antitussive (Hosseinzadeh and Ghenaati, 2006 ▶) and anti-inflammatory (Boskabady et al., 2012 ▶; Hosseinzadeh and Younesi, 2002 ▶; Mousavi and Bathaie, 2011 ▶) effects of saffron have also been reported. Moreover, previous reports showed the relaxant effect of saffron on tracheal smooth muscle (Boskabady and Aslani, 2006 ▶). The inhibitory effect of the plant on histamine (H_1_) receptor (Boskabady et al., 2010b ▶) and its stimulatory effect on β-adrenoceptors (Nemati et al., 2008 ▶) have also been documented. 

Therefore, according to known properties of saffron and pathogenesis of asthma, the effect of the hydroalcoholic extract of *C. sativus* on total blood elements and differential white blood cells (WBC) count in peripheral blood of sensitized rats was evaluated in this study.

## Materials and Methods

**Plant material and preparation of the extract**

Saffron was harvested from saffron farms of Boshrooyeh (northeast of Iran) and the plant was identified by botanists in the herbarium of Ferdowsi University of Mashhad. The specimen number of the plant is 293-0303-1. 

Hydroalcoholic extract was prepared with 10 g of its ground petal stigma and 400 mL of 70% aqueous-alcoholic solution in a Soxhlet extractor for 14 h. The prepared extract was concentrated to 100 mL with a rotatory evaporator in low pressure and filtered through a 0.2-mm filter to be sterilized. The resulting extract was concentrated under reduced pressure and stored at −20 °C until being used. The extract was dissolved in saline in order to arrive at desired concentrations and was then applied.

**Study design**

The experiments were performed using 40 male Wistar rats weighing approximately 200–250 g and were randomly divided into five groups (n=8 for each group) as follows: 

1. Control group (not sensitized, group C).

2. Sensitized with ovalbumin (OA) alone (group asthma).

3. Sensitized and treated with 50 mg/kg extract of* C. sativus* post sensitization during 32 days (group asthma+50EX).

4. Sensitized and treated with 100 mg/kg extract of* C. sativus* post sensitization during 32 days (group asthma+100EX).

5. Sensitized and treated with 200 mg/kg extract of* C. sativus* post sensitization during 32 days (group asthma+200EX).

**Animal sensitization and animal groups**

The animals were kept in a 22±2 °C temperature with a 12 h light/dark cycle and fed with standard diet and tap drinking water. The animals were acclimatized for at least 7 days before use in experiments and then, animals were sensitized to OA according the method described previously (Boskabady and Adel-Kardan, 1999 ▶; Boskabady and Ziaei, 2003 ▶). Briefly, rats were sensitized to 1 mg OA (Sigma Chemical Ltd, UK) and 50 mg Al(OH)_3_ dissolved in 0.5 ml saline i.p. One week later, they were given 0.02 mg OA and 50 mg Al(OH)_3_ dissolved in 0.5 ml saline i.p. as a booster dose. From day 14, sensitized animals were exposed to an aerosol of 4% OA for 18±1 days, 5 min daily. 

The aerosol was administered in a closed chamber, dimensions 30×20×20 cm^3^ using a nebulizer (CX3, Omron Healthcare Europe B.V., the Netherlands). Control animals were treated similarly but saline was used instead of OA solution. Treated animals received different concentrations of saffron extract twice a week as intraperitoneal injection simultaneously for sensitization during 32 days. The study was performed in the Department of Biology, Faculty of Science, Mashhad Branch, Islamic Azad University, Iran, and was approved by the ethical committee of the same institute.

**Blood cells count**

At the end of the experiment period, the rats were anesthetized by intraperitoneal injection of chloral hydrate (400 mg/kg). With a EDTA-coated syringe, five ml blood sample was taken by cardiac puncture immediately after anesthesia and exposing the animals chest. The leukocyte count was determined on blood samples diluted 1:10 in Turk solution by means of a Neubauer’s hemocytometer. The Turk solution consisted of 1 mL of glacial acetic acid, 1 mL of Gentian Violet Solution 1% and 100 mL distilled water. To avoid aggregation of white blood cells, fresh blood always maintained on ice. Instantly, selected smears were fixed with methanol and stained with Giemsa’s solution, and then used for a differential count of the white blood cells. According to staining and morphological criteria, differential cell analysis was done under the light microscope by counting 100 cells, and the percentage of each cell type was calculated. 

The erythrocyte number (RBC) was counted in a Neubauer’s hemocytometer after the sample was diluted (1:200) in saline solution. For determining the platelet count, whole blood was diluted with 1% ammonium oxalate solution. The standard dilution for platelet counts was 1:100. The dilution was mixed well and incubated to permit lysis of the erythrocytes. Following the incubation period, the dilution was mounted on a hemacytometer. The cells were allowed to settle and then were counted in a specific area of the hemacytometer chamber under the light microscope.

**Statistical analysis**

All data are expressed as mean±SEM. Comparisons were performed using one-way ANOVA with SPSS software. p-values less than 0.05 were considered to be statistically signiﬁcant.

## Results

**Total and differential WBC count**

**Figure 1:**
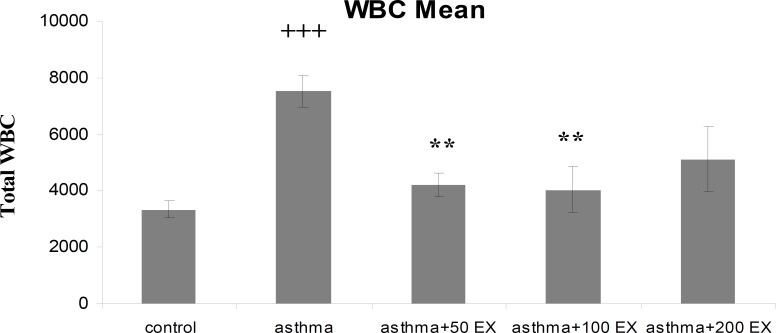
Total number of blood WBC in control, sensitized (asthma), and sensitized treated with three concentrations of the extract (asthma+50Ex, asthma+100Ex and asthma+200Ex). Data are expressed as mean±SEM. (n = 8). +++p<0.001, versus control group. **p<0.01, versus asthma group

**Figure 2:**
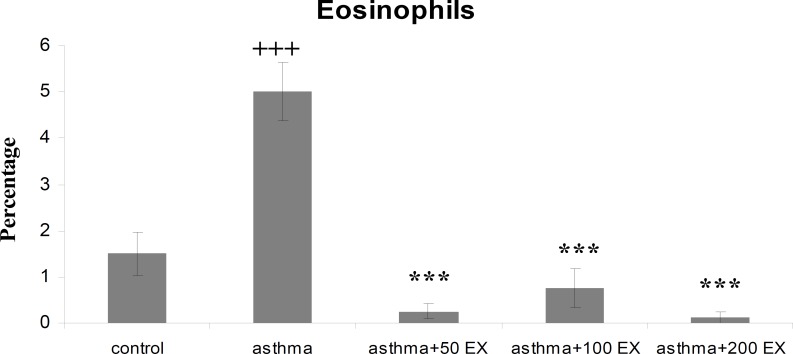
Percentage of blood eosinophils in control, sensitized (asthma), and sensitized treated with three concentrations of the extract (asthma+50Ex, asthma+100Ex and asthma+200Ex). Data are expressed as mean±SEM. (n = 8). +++p<0.001, versus control group. ***p<0.01, versus asthma group

**Figure 3:**
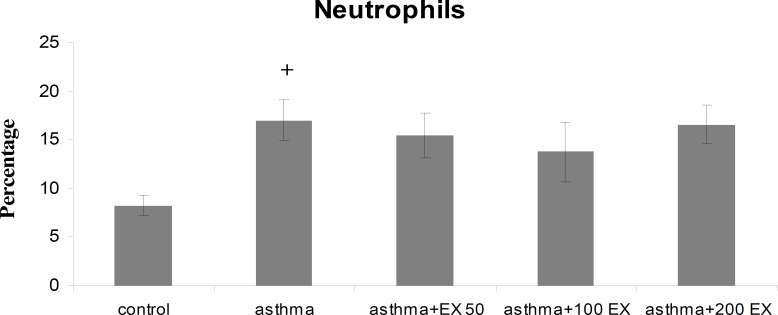
Percentage of blood neutrophils in control, sensitized (asthma), and sensitized treated with three concentrations of the extract (asthma+50Ex, asthma+100Ex and asthma+200Ex). Data are expressed as mean±SEM. (n = 8). +p<0.05, versus control group

**Figure 4:**
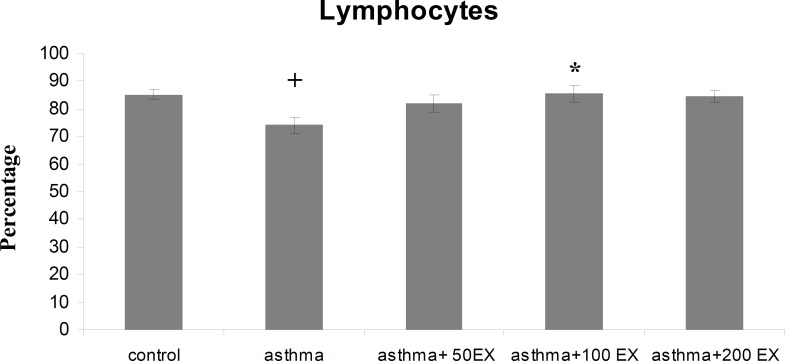
Percentage of blood lymphocytes in control, sensitized (asthma), and sensitized treated with three concentrations of the extract (asthma+50Ex, asthma+100Ex and asthma+200Ex). Data are expressed as mean±SEM. (n = 8). +p<0.05, versus control group. *p<0.05, versus asthma group

Total WBC count and the percentage of eosinophil and neutrophil in blood of group asthma were signiﬁcantly higher than those of group C (p<0.001 for total WBC count and eosinophil and p<0.05 for neutrophil) (Figures 1 to 3). The percentage of lymphocyte in group asthma was lower than group C (p<0.05) (Figure 4). In sensitized animals treated with different concentrations of saffron, total WBC was decreased compared with asthmatic group. These reductions were significant in the first two concentrations of extract (p<0.01 for groups of asthma+50EX and asthma+100EX) (Figure 1). Treatment with different concentrations of extract caused significant decreases in eosinophil percentage (p<0.001 for all groups) (Figure 2). Compared with sensitized animals, there was a reduction in percentage of neutrophils in animal groups treated with different concentrations of extract which was not significant (Figure 3). The percentage of lymphocyte in groups received the extract, were higher than group asthma (p<0.05 in asthma+100EX) (Figure 4).

**RBC count**

**Figure 5:**
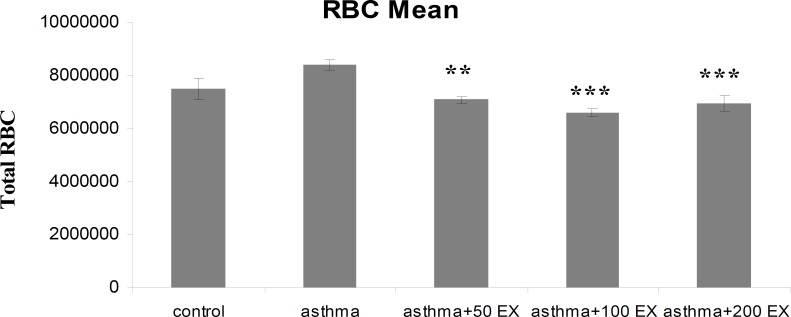
Total number of blood RBC in control, sensitized (asthma), and sensitized treated with three concentrations of the extract (asthma+50Ex, asthma+100Ex and asthma+200Ex). Data are expressed as mean±SEM. (n = 8). **p<0.01 and ***p<0.01 versus asthma group

In group asthma, mean number of RBC increased compared with group C, but, this increase was not significant. However, RBC count in sensitized animals treated with different concentrations of saffron was reduced significantly (p<0.01 to p<0.001) (Figure 5).

**Platelet count**

**Figure 6:**
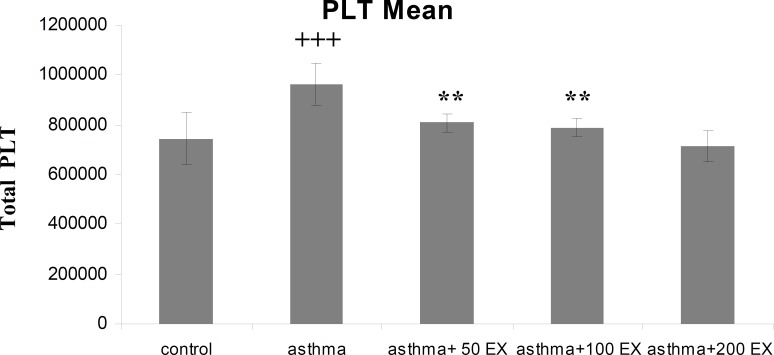
Total number of blood platelet count in control, sensitized (asthma), and sensitized treated with three concentrations of the extract (asthma+50Ex, asthma+100Ex and asthma+200Ex). Data are expressed as mean±SEM. (n = 8). +++p<0.001, versus control group. **p<0.01 versus asthma group

The mean platelet count in group asthma was higher than group C (p<0.001). In sensitized animals which received the extract of saffron a reduction was observed in mean platelet number, which was significant in the first two concentrations of extract compared with group asthma (p<0.01 for groups asthma+50EX and asthma+100EX) (Figure 6). 

## Discussion

The results of this study showed increased total WBC, eosinophil, and neutrophil, but reduced lymphocyte count in sensitized animals compared with control ones. These data are along with the results obtained by Adamko et al. based on the increase in the number of mast cells, eosinophils, lymphocytes, neutrophils, and macrophages in mucosal layer of airway tract in asthmatic patients (Adamko et al., 2003 ▶). The increased total WBC and eosinophil count in sensitized animals have already been shown in previous studies that can confirm the sensitization of the rats in the present study (Boskabady et al., 2010a ▶; Boskabady et al., 2010b ▶). 

Asthma is characterized by an inflammatory state resulting in activation of lung tissue cells and attraction and infiltration of leukocytes from the blood. The accumulation of eosinophilic leukocytes is a prominent feature of inflammatory reactions that occurs in allergic asthma. The increase in number of eosinophils is important since it correlates in time with an increase in bronchial hyper-responsiveness (Bloemen et al., 2007 ▶). They can be primed by a number of factors, such as IL-3, IL-5, chemokines, and platelet activating factor (PAF), resulting in inflammatory mediator release (Prussin and Metcalfe, 2006 ▶) such as major basic protein (MBP), eosinophil cationic protein (ECP), eosinophil-derived neurotoxin (EDN), and eosinophil peroxidase (EP) (Pearlman, 1999 ▶). Together, they can cause substantial damage to airway endothelial cells and extracellular matrix (ECM).

Not only eosinophilic inflammation but also neutrophilic inflammation may play roles in the pathogenesis of asthma. They can produce a wide range of products, including lipids (LTA4, LTB4, PAF, thromboxane (TX) A2), cytokines (IL-1β, IL-6, TNF-α, TGF-β, CXCL8), proteases (elastase, collagenase, matrix metalloproteinase (MMP) 9), microbicidal products (lactoferrin, myeloperoxidase, lysozyme), reactive oxygen intermediates (superoxide, hydrogen peroxide, OH^−^), and nitric oxide (NO). Neutrophil products can cause airway narrowing, increased mucus secretion and increased airway hyper-responsiveness (Bloemen et al., 2007 ▶).

T cells, especially T helper 2 (Th2) lymphocytes, are important during the whole allergic cascade. However, a co-operative interaction between Th2 and T helper 1 (Th1) cytokines is involved in the pathogenesis of asthma. Th2 cells secrete cytokines, such as IL-3, IL-4, IL-5, IL-6, IL-9, IL-10, and IL-13, which exert a whole range of inflammatory effects. During response to environmental antigens in atopic subjects, Th2 cytokines are responsible for the production of IgE. In later stages of asthma, these cytokines are responsible for ongoing inflammation. Th1 cells secrete cytokines, such as IFN-γ, IL-2, IL-12, IL-18, TNF-α, and TNF-β. Several studies show elevated levels of pro-inflammatory cytokines in bronchoalveolar lavage fluid and blood of asthmatic patients and sensitized animals (Boskabady et al., 2012 ▶; Kim et al., 2003 ▶).

Pretreatment of sensitized animals with the extract of *C. sativus* caused significant decrease in total and differential inflammatory cells. The extract especially reduced eosinophil percentage in all concentrations. Since the main pathophysiological feature of asthma is airway inflammation, treatment of this disease should improve this phenomenon, so our findings show possible inhibitory effect of the saffron extract on inflammatory cells in sensitized animals. Moreover, these results confirm the anti-inflammatory property of *C. sativus *(Boskabady et al., 2012 ▶; Hosseinzadeh and Younesi, 2002 ▶). RBC count are increased in asthmatic patients due to narrowed and inflamed airway tracts thus air flow decreases and less oxygen reaches the lungs, moreover, asthma limits oxygen-carrying capacity of RBCs which makes the bone marrow produce more RBCs. Although increase of RBC count in group asthma was not significant compared with control group, pretreatment with different concentrations of *C. sativus* inhibited elevation of RBC count in sensitized animals. This inhibitory effect of extract is in favor of anti-inflammatory effect of extract which maybe subsequently improved hyper-responsiveness and function of respiratory airways. 

Our results indicate increase in platelet number of sensitized animals, as in previous studies also platelet activation has been demonstrated in different inflammatory lung diseases including asthma (Averill et al., 1992 ▶; Kowal et al., 2006 ▶). Release of mediators contained in all types of platelet granules such as PAF and TGF-β can occur after activation that helps to improve inflammation process during asthma. *C. Sativs* in different concentrations, prevented the increase of platelet numbers in sensitized animals which can support the observations made in former studies to express the anti-inflammatory effect of saffron (Boskabady et al., 2012 ▶; Hosseinzadeh and Younesi, 2002 ▶). 

Taken together these results demonstrated the preventive effect of the extract of *C. sativus* on WBC count, eosinophil percentage, and platelet number in the blood of sensitized rats which can indicate a prophylactic effect for the extract of the plant on asthma.
